# Viable *Ednra*^*Y129F*^ mice feature human mandibulofacial dysostosis with alopecia (MFDA) syndrome due to the homologue mutation

**DOI:** 10.1007/s00335-016-9664-5

**Published:** 2016-09-26

**Authors:** Sibylle Sabrautzki, Michael A. Sandholzer, Bettina Lorenz-Depiereux, Robert Brommage, Gerhard Przemeck, Ingrid L. Vargas Panesso, Alexandra Vernaleken, Lillian Garrett, Katharina Baron, Ali O. Yildirim, Jan Rozman, Birgit Rathkolb, Christine Gau, Wolfgang Hans, Sabine M. Hoelter, Susan Marschall, Claudia Stoeger, Lore Becker, Helmut Fuchs, Valerie Gailus-Durner, Martin Klingenspor, Thomas Klopstock, Christoph Lengger, Leuchtenberger Stefanie, Eckhard Wolf, Tim M. Strom, Wolfgang Wurst, Martin Hrabě de Angelis

**Affiliations:** 1 Institute of Experimental Genetics and German Mouse Clinic, Helmholtz Zentrum München, German Research Center for Environmental Health (GmbH), Ingolstädter Landstr.1, 85764 Neuherberg, Germany; 2 Research Unit Comparative Medicine, Helmholtz Zentrum München, German Research Center for Environmental Health (GmbH), Ingolstädter Landstr.1, 85764 Neuherberg, Germany; 3 Comprehensive Pneumology Center, Institute of Lung Biology and Disease, Helmholtz Zentrum München, Ingolstädter Landstr.1, 85764 Neuherberg, Germany; 4 Institute of Human Genetics, Helmholtz Zentrum München, German Research Center for Environmental Health (GmbH), Ingolstädter Landstr.1, 85764 Neuherberg, Germany; 5 German Center for Diabetes Research (DZD), Neuherberg, Germany; 6 Department of Neurology, Friedrich-Baur-Institut, University Hospital of LMU Munich, Ziemssenstr. 1, 80336 Munich, Germany; 7 German Center for Vertigo and Balance Disorders, 81377 Munich, Germany; 8 Institute of Developmental Genetics, Helmholtz Zentrum München, German Research Center for Environmental Health (GmbH), Ingolstädter Landstr.1, 85764 Neuherberg, Germany; 9 Ludwig-Boltzmann Gesellschaft GmbH, Ludwig-Boltzmann Institut for Clinical-Forensic Imaging (LBI CFI), BioTechMed Graz, Universitätsplatz 4/2, 8010 Graz, Austria; 10 Molecular Nutritional Medicine, Else Kröner-Fresenius Center and ZIEL Research Center for Nutrition and Food Science, Technische Universität München, Gregor-Mendel-Str. 2, 85350 Freising-Weihenstephan, Germany; 11 Gene Center of the Ludwig-Maximilians-Universität München, Chair for Molecular Animal Breeding and Biotechnology, Ludwig-Maximilians-Universität München, Feodor-Lynen-Str. 25, 81377 Munich, Germany; 12 Munich Cluster for System Neurology (SyNergy), Adolf-Butenandt-Institut, Ludwig-Maximilians-Universität München, Schillerstr. 44, 80336 Munich, Germany; 13 Chair of Developmental Genetics at Technische Universität München-Weihenstephan, Helmholtz Zentrum München, Ingolstädter Landstr.1, 85764 Neuherberg, Germany; 14 German Center for Neurodegenerative Diseases (DZNE) Site Munich, Feodor-Lynen-Str. 17, 81377 Munich, Germany; 15 Institut für Humangenetik, Klinikum rechts der Isar der Technischen Universität München, Trogerstr. 32, 81675 Munich, Germany; 16 Lehrstuhl für Experimentelle Genetik, Technische Universität München, 85350 Freising-Weihenstephan, Germany

## Abstract

Animal models resembling human mutations are valuable tools to research the features of complex human craniofacial syndromes. This is the first report on a viable dominant mouse model carrying a non-synonymous sequence variation within the endothelin receptor type A gene (*Ednra* c.386A>T, p.Tyr129Phe) derived by an ENU mutagenesis program. The identical amino acid substitution was reported recently as disease causing in three individuals with the mandibulofacial dysostosis with alopecia (MFDA, OMIM 616367) syndrome. We performed standardized phenotyping of wild-type, heterozygous, and homozygous *Ednra* ^Y129F^ mice within the German Mouse Clinic. Mutant mice mimic the craniofacial phenotypes of jaw dysplasia, micrognathia, dysplastic temporomandibular joints, auricular dysmorphism, and missing of the squamosal zygomatic process as described for MFDA-affected individuals. As observed in MFDA-affected individuals, mutant *Ednra* ^Y129F^ mice exhibit hearing impairment in line with strong abnormalities of the ossicles and further, reduction of some lung volumetric parameters. In general, heterozygous and homozygous mice demonstrated inter-individual diversity of expression of the craniofacial phenotypes as observed in MFDA patients but without showing any cleft palates, eyelid defects, or alopecia. Mutant *Ednra* ^Y129F^ mice represent a valuable viable model for complex human syndromes of the first and second pharyngeal arches and for further studies and analysis of impaired endothelin 1 (EDN1)–endothelin receptor type A (EDNRA) signaling. Above all, *Ednra* ^Y129F^ mice model the recently published human MFDA syndrome and may be helpful for further disease understanding and development of therapeutic interventions.

**Electronic supplementary material:**

The online version of this article (doi:10.1007/s00335-016-9664-5) contains supplementary material, which is available to authorized users.

## Introduction

Studies in humans and in animal models have indicated an essential role of the endothelin 1 (EDN1,2)–endothelin receptor type A (EDNRA) signaling in normal craniofacial development. Within the large family of syndromes of the first and second pharyngeal arches, craniofacial deformities in humans may occur as single characteristic without any further organ anomalies, e.g., observed in auriculocondylar syndrome (ACS, OMIM 602483, 614669, and 615706) due to disruption of the EDNRA signaling pathway (Clouthier et al. 2013; Gordon et al. 2013) or may occur with further organ anomalies, e.g., observed in oculo-auriculo-vertebral spectrum (OAVS, OMIM 164210). It was described for mouse embryos and zebrafish larvae that Edn1 is required for the formation of ventral arch cartilage and bones (dentary, hyoid, thyroid, and tympanic ring bone), while the formation of more proximal cartilage and bones such as maxilla, palatine, and pterygoid is inhibited (Clouthier et al. 2010). Thus, in ACS as a well-characterized rare craniofacial disorder affecting early neural crest cell (NCC) development within the first and second pharyngeal arches, clinical characteristics manifest usually within structures developed from differentiated NCCs like the bones of the upper and lower jaw, the ossicles, the outer ear, and the neck (Passos-Bueno et al. 2009; Ruest and Clouthier 2009). In addition to several other genes, mutations of *EDN1* were reported to cause auriculocondylar syndrome 3 in patients (ARCND3, OMIM 615706) (Gordon et al. 2013). In mouse models, mutations of genes of the endothelin-1 pathway led to craniofacial symptomatology, as mice harboring a targeted mutation either in *Edn1* (MGI:95283), *Ednra* (MGI:105923), or the endothelin-converting enzyme-1 gene (*Ece1;* MGI:1101357) coding for an enzyme responsible for the processing of inactive big-EDN1 to active EDN1 were born with craniofacial and cardiovascular malformations (Kurihara et al. 1994; Clouthier et al. 1998; Yanagisawa et al. 1998; Kitazawa et al. 2011). Examining abnormalities associated with disruptions in these mouse genes was limited so far as knockouts exhibited embryonic or neonatal lethality.

Recently, sequence analysis of one patient with severe craniofacial abnormalities, alopecia, ear malformations, and conductive hearing loss in line with developmental delay, originally described for Johnson–McMillin syndrome (Cushman et al. 2005), revealed that these abnormalities were due to a de novo missense mutation within the *EDNRA* gene as additionally isolated in two other patients. Thus, a new syndrome was suggested as mandibulofacial dysostosis with alopecia (MFDA, OMIM 616367) (Gordon et al. 2015).

Here, we describe a new viable *Ednra* ^Y129FMhda^ mouse model carrying the homologue point mutation of the three patients reported with MFDA (Gordon et al. 2015). The model was derived by the Munich *N*-ethyl-*N*-nitrosourea (ENU) mutagenesis project used for more than a decade as a forward genetic approach to generate mutant mouse models for inherited human diseases (Hrabě de Angelis et al. 2000; Sabrautzki et al. 2012). Using the standardized phenotyping platform of the German Mouse Clinic (GMC) (Gailus-Durner et al. 2009; Fuchs et al. 2012), we observed a diversity of dysmorphological, otolaryngeal, and lung phenotypes by comparison of adult heterozygous (*Ednra* ^Y129F/+^) and homozygous (*Ednra* ^Y129F/Y129F^) mice with their wild-type littermates (*Ednra* ^+/+^). Our results may contribute to reveal single-gene contribution in MFDA and may be helpful to develop a treatment strategy for patients (Gordon et al. 2015).

## Materials and methods

### ENU mutagenesis and mice

ENU mutagenesis was performed on the pure inbred C3HeB/FeJ (C3H) mouse strain purchased originally from the Jackson Laboratory (Bar Harbour, Maine) as previously described (Aigner et al. 2011; Sabrautzki et al. 2012). The mice were housed and handled according to the federal animal welfare guidelines and the responsible authority of Upper Bavaria approved all animal studies (Reference Numbers 55.2-1-54-2532-78-06 and 55.2-1-54-2532-144-10). The mouse line was given the internal lab code AEA001 (**a**bnormal **ea**r #1) according to the big bat-like ears. Heterozygous intercrosses led to homozygous offspring in a Mendelian ratio that were smaller than heterozygous littermates and showed bigger ears. After the causative mutation was isolated, the mouse line was given the official name *Ednra* ^Y129FMhda^.

### Phenotyping in the GMC

Phenotyping was performed in the GMC according to the standardized IMPReSS protocols (www.mousephenotype.org). Depending on the individual screens, cohorts of 9–15 *Ednra* ^Y129F/+^, *Ednra* ^Y129F/Y129F^, and *Ednra* ^+/+^ mice per gender were analyzed in the age of 9–16 weeks. Phenotyping was performed by a total of fourteen independent screens (Fuchs et al. 2011). A summary of all phenotyping protocols is provided in Table S1.

#### pQCT measurement

Femur midshafts and distal metaphyses of *Ednra* ^Y129F/+^ mice were scanned by pQCT (Norland Stratec XCT, Stratec Medizintechnik GmbH, Pforzheim, Germany) at 3, 6, 9, and 12 months of age for the analyses of total bone area, bone mineral content (BMC), and bone mineral density (BMD) without distinguishing between cortical and trabecular bone. Two pQCT scan slices were obtained at each site, with voxel dimensions of 70 × 70 × 500 µm providing data for 1 mm of bone length.

#### Micro-CT analysis and cephalometric measurement

**Fig. 1:**
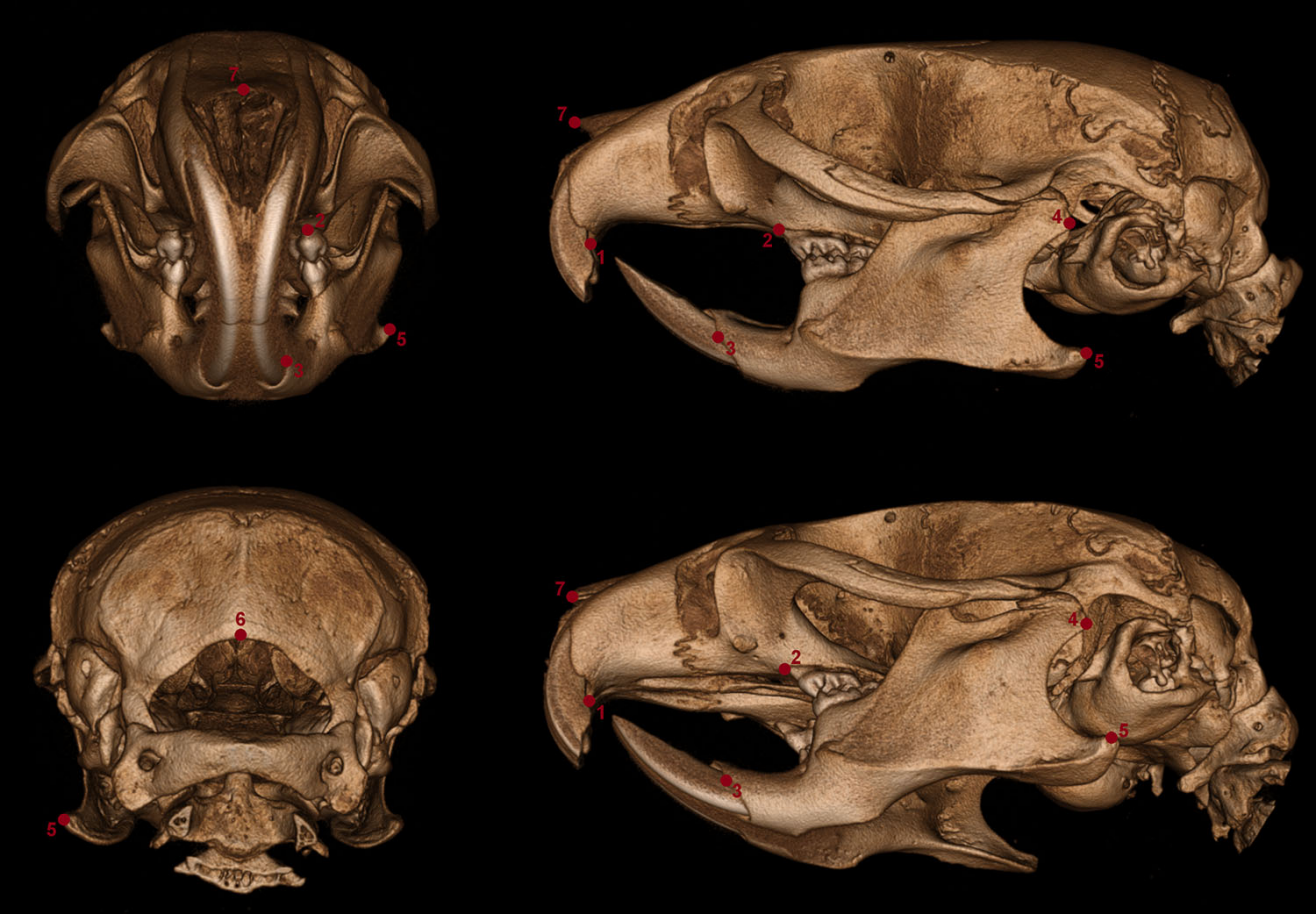
Landmarks of cephalometric/mandibular measurement

Adult skulls (26- to 30-week-old mice) were imaged using a SkyScan 1176 in vivo CT (Bruker micro-CT N.V., Kontich, Belgium) at 9 µm pixel resolution using 100 kV voltage, 100 mA current, and a 0.5 mm aluminum filter. The resulting slices were reconstructed with SkyScan’s NSRECON package using uniform attenuation coefficient data as previously described (Sabrautzki et al. 2013; Sandholzer et al. 2014). In total, eight datasets were evaluated by two experienced osteologists using the distance measurement tool of 3DSlicer 4.5 (available online: www.slicer.org). Cranial and mandibular reference points on the bone surface (Fig. 1) were selected based on previously described landmarks (De Carlos et al. 2011). Soft-tissue contrast enhancement was reached by staining with an Iodine–Potassium Iodine solution for 2 weeks, with the solution exchanged every 48 h.

#### Whole mount analysis of middle and inner ears

Samples were fixed in paraformaldehyde, dehydrated in ethanol series, and made translucent using methyl-salicylate. Afterwards, the translucent inner ears were observed and photographed using top and bottom illumination under the microscope.

#### Lung analysis

For analysis of pulmonary function, five *Ednra* ^+/+^, six *Ednra* ^Y129F/+,^ and six *Ednra* ^Y129F/Y129F^ female mice were used. The anesthetized mice were tracheotomized and ventilated during the experiment. Briefly, a FinePointe RC system (Buxco, Wilmington, USA) was used to measure dynamic lung compliance (Cdyn) and resistance. Afterwards, the mice were transferred to a Biosystem XA forced maneuvers system (Buxco, Wilmington, USA) to measure forced expiratory volume in 0.1 s (FEV0.1), forced vital capacity (FVC), functional residual capacity (FRC), total lung capacity (TLC), tidal volume (TV), inspiratory capacity (IC), expiratory reserve volume (ERV), vital capacity (VC), residual volume (RV), and static lung compliance (Cchord).

#### Statistical analyses

Statistical analyses were done using *t test*, Wilcoxon rank-sum test, linear models, or ANOVAs, depending on the assumed distribution of the parameter and the questions addressed to the data. A *P* value <0.05 has been used as the level of significance; a correction for multiple testing has not been performed.

#### Genetic mapping

For genetic mapping, phenotypically heterozygous mice were backcrossed with C57BL/6J (B6) mice, and DNA was extracted from tail tips from 44 mutant and 37 wild-type B6–C3H hybrid mice for linkage analysis as described previously (Aigner et al. 2011). Genotyping was performed by single-nucleotide polymorphism (SNP) analysis using MassExtend (MALDI-TOF MS genotyping system) (Sequenom, San Diego, CA, USA) as previously described (Sabrautzki et al. 2012).

#### Exome sequencing

DNA extraction from the spleens was performed using ProteinaseK, RNaseA, CellLysis Solution, Protein Precipitation Solution, and DNA Hydration Solution from Qiagen according to the manufacturer’s manual (Qiagen, Venlo,the Netherlands). In-solution targeted enrichment of exonic sequences from both the phenotypically mutant and the control wild-type littermate mouse using the SureSelectXT Mouse All Exon kit (Agilent, Santa Clara, CA, USA) was performed. Libraries were sequenced as 100 bp paired-end runs on a HiSeq 2000 system (Illumina, San Diego, CA, USA). Read alignment to mouse genome assembly mm9 was done with Burrows-Wheeler Aligner (BWA, version 0.5.9), and a total of 10 and 9.2 Gb of mapped sequence data corresponding to an average coverage of 120× (>95 % of the target being covered >20×) and 113× for the mutant and control, respectively, were yielded. Single-nucleotide variants (SNVs) and small insertions and deletions (indels) were detected with SAMtools (v. 0.1.7).

## Results

### Genotypic identification of a new ENU-derived mouse model

Using the sources of the large-scale genome-wide Munich ENU mutagenesis project (MEP), a mouse line with craniofacial and ear abnormalities was identified (AEA001, abnormal ear #1), thereby showing an autosomal dominant mode of inheritance. Linkage analysis gave a highest Chi square for a region on chromosome eight between the genomic markers rs13479782 and rs13479952 (NCBI137/mm9_chr8:60,521,202-103,433,460, ~43 Mb, UCSC). Due to the high number of candidate genes within this large region, we performed whole exome sequencing for mutation detection. By comparing and filtering the sequencing data as described previously (Sabrautzki et al. 2013; Diener et al. 2016), we obtained 11 candidate SNVs. Three candidate SNVs were located within the candidate linkage interval on mouse chromosome 8. Although linkage data were available, we decided to analyze all 11 variants of interest within a cohort of 10 wild-type and 16 mutant mice by capillary sequencing. Only one SNV on mouse chromosome 8, genomic position NCBI137/mm9_chr8:80,243,961, segregated in all mutant mice analyzed with the phenotype. This private SNV was a heterozygous non-synonymous sequence variation within the *Ednra* gene (NM_010332.2:c.386A>T, NP_034462.1:p.Tyr129Phe). Thus, the AEA001 mouse line was renamed as *Ednra* ^Y129FMhda^. Recently, in humans the non-synonymous sequence variation within the *EDNRA* gene at the corresponding position (NM_001957.3:c.386A>T, NP_001948.1:p.Tyr129Phe) was published as disease causing in three unrelated individuals with MFDA (OMIM 616367) (Gordon et al. 2015).

### Morphological changes in Ednra^Y129F^ mice

**Fig. 2:**
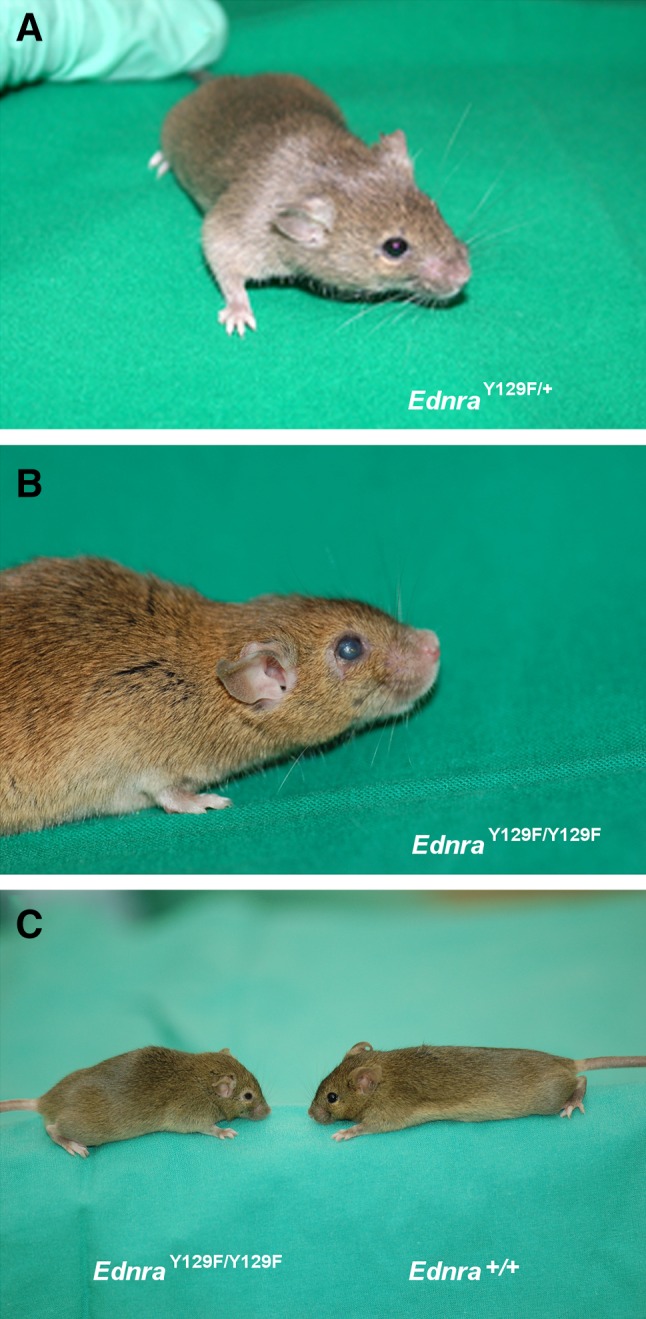
Visible ear and eye abnormalities of *Ednra* ^Y129F/+^ and *Ednra* ^Y129F/Y129F^ mice

Mutant *Ednra* ^Y129F^ mice showed craniofacial abnormalities as short snout, round facial, and shortened head appearance, further prominent cheeks, micrognathia, and significantly malformed big bat-like ears (Fig. 2a, b, c). The low-settled ears had a tapered helix, and the eyes of some mice seemed to be enlarged. *Ednra* ^Y129F/Y129F^ mice were viable and fertile but displayed a visibly decreased body size compared to *Ednra* ^+/+^ and *Ednra* ^Y129F/+^ mice with shortening and flattening of the skull in some but not all *Ednra* ^Y129F/Y129F^ mice. We observed a general inter-individual variance in the severity of phenotype expression among the mutant genotypes.

### Micro-CT imaging of craniofacial phenotypes

**Fig. 3:**
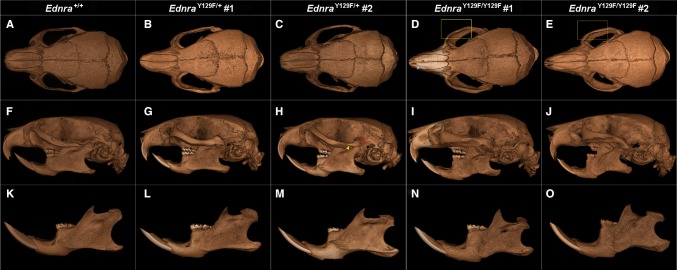
Micro-CT analysis of the dorsal (**a**–**e**), left lateral view (**f**–**j**) of the skull and lateral view of the mandible (**k**–**o**)

Three-dimensional micro-CT analysis and measurements revealed bones of the skull misshaped or underdeveloped in *Ednra* ^Y129F/+^ and *Ednra* ^Y129F/Y129F^ mice (Fig. 3, Movies M1–M3). The dorsal view depicted broadening of the sagittal, coronal, and anterior lambdoid sutures of an *Ednra* ^Y129F/Y129F^ mouse (Fig. 3d) and broadened zygomatic bones without any sutures in both *Ednra* ^Y129F/Y129F^ mice (Fig. 3d, e). In the lateral view, the absence of the zygomatic process of the squamosal bone was obvious in both *Ednra* ^Y129F/Y129F^ mice (Fig. 3i, j), as also seen in *Ednra* ^Y129F/+^ mouse #1 (Fig. 3g). In these three mice (Fig. 3g, i, j), the zygomatic bone, connecting the malar and squamosal zygomatic processes, appeared fused without any suture (Movie M1). In addition, the shape of the zygomatic bone was abnormal in these mice when compared to the *Ednra* ^+/+^ mouse (Fig. 3f). In *Ednra* ^Y129F/+^ mouse #2 (Fig. 3h), the squamosal part of the zygomatic process appeared to be present, as indicated by the existence of a suture but with a gap to the zygomatic bone. Similar to the findings in MFDA patients, we observed morphological changes of the orbit in mutant *Ednra* ^Y129F^ mice. The orbit appeared round and with thickened malar zygomatic bone in *Ednra* ^Y129F/Y129F^ mice, while in the *Ednra* ^+/+^ mouse the orbit had an oval appearance. No dental abnormalities or cleft palates were found in mutant *Ednra* ^Y129F^ mice. A malocclusion of molar teeth was observed in *Ednra* ^Y129F/Y129F^ mouse #2 (Fig. 3j) and a minor malocclusion in *Ednra* ^Y129F/+^ mouse #2 (Fig. 3h). Altogether, the bone abnormalities of the skull in mutant mice were highly similar to the changes found in clinical CT scans of MFDA patients (Gordon et al. 2015).

Jaw dysplasia of the mandibular condyloid process was visible in all mutant mice investigated (Fig. 3l, m, n, o; Movie M2). In accordance with observations in MFDA patients, *Ednra* ^Y129F/+^ and *Ednra* ^Y129F/Y129F^ mice showed micrognathia by mandibular shortening of the diastema (8.1 % in three *Ednra* ^Y129F/+^ and 14.6 % in two *Ednra* ^Y129F/Y129F^ mice; Table 1). Furthermore, an abnormality of the temporomandibular joint in mutant mice was evident (Movie M3).

**Table 1:** Craniometric and mandibular measurements of eight *Ednra* mice (*Ednra* ^+/+^ *n* = 3, *Ednra* ^Y129/+^ *n* = 3, *Ednra* ^Y129F/Y129F^ *n* = 2)

ID	Genotype	A [1–2]	B [3–4]	C [3–5]	D [4–5]	E [6–7]	Ratio C/A
1	*Ednra* ^+/+^	6.19	11.8	11.8	3.6	23.9	1.91
2	*Ednra* ^+/+^	6.11	11.5	11.4	3.5	23.1	1.87
3	*Ednra* ^+/+^	6.23	10.9	11.1	3.4	23.9	1.78
4	*Ednra* ^Y129F/Y129F^	6.61	11.3	11.1	3.2	22.1	1.68
5	*Ednra* ^Y129F/+^	6.25	11	10.7	3.1	22	1.71
6	*Ednra* ^Y129F/+^	6.35	11.1	10.9	3	22.4	1.72
7	*Ednra* ^Y129F/Y129F^	6.96	10.9	11.2	2.6	23.3	1.61
8	*Ednra* ^Y129F/Y129F^	6.82	10.3	10.6	2.5	22.6	1.55
Mean C/A ratio		*Ednra* ^+/+^ 1.85		*Ednra* ^Y129F/+^ 1.70		*Ednra* ^Y129F/Y129F^1.58	

Measurement points: (1) palatinal intra-incisor-alveolar point, (2) palatinal-molar-alveolar point, (3) buccal incisor-alveolar point, (4) most inferior point of the *Processus condylaris*, (5) most superior posterior point of the *Processus angularis*, (6) most dorsal point of the *Foramen magnum* at the *Os occipitale*, and (7) most caudal point of the *Os nasale*. Mean distance in mm

### Hearing impairment

**Fig. 4:**
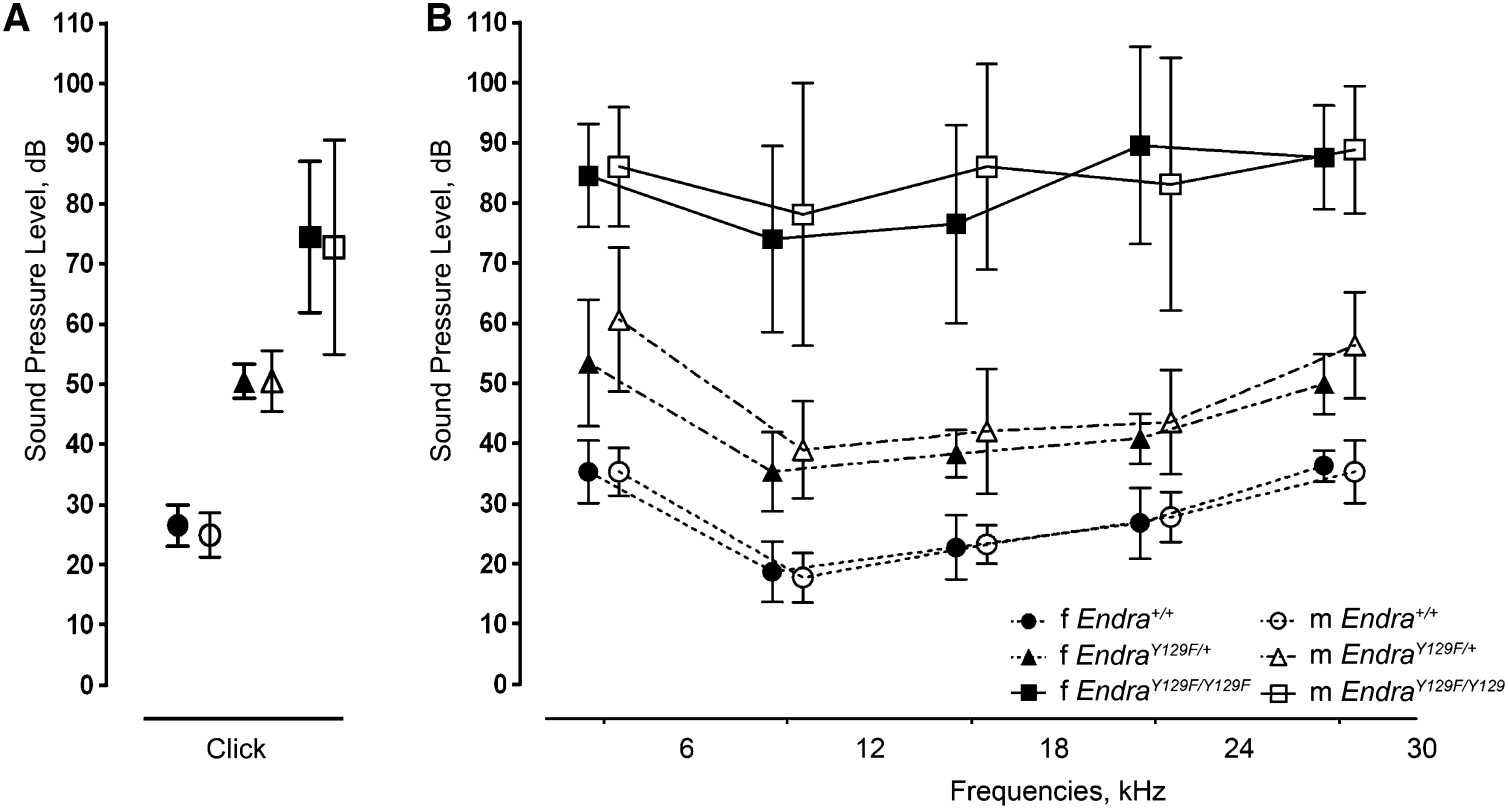
ABR thresholds for click and tone-evoked potentials in *Ednra* ^+/+^, *Ednra* ^Y129F/+^, and *Ednra* ^Y129F/Y129F^ mice

In line with the conductive hearing loss of all MFDA patients (Gordon et al. 2015), mild hearing impairment was observed in *Ednra* ^Y129F/+^ mice. An even stronger hearing loss in *Ednra* ^Y129F/Y129F^ mice was demonstrated by auditory brainstem response ABR analysis with thresholds increased by 25 and 50 dB, respectively, compared to the thresholds obtained for *Ednra* ^+/+^ mice for both click (Fig. 4a) and pure tone stimuli (Fig. 4b). Conductive hearing impairment for mutant mice was further affirmed by behavioral tests. Acoustic startle reactivity was significantly decreased (*P* < 0.001) in *Ednra* ^*Y129F/Y129F*^ mice compared to *Ednra* ^+/+^ and *Ednra* ^Y129F/+^ mice (Fig. S1). Moreover, there was decreased prepulse inhibition (PPI) in mutant mice, with a significant reduction in *Ednra* ^Y129F/Y129F^ mice at all prepulse intensities (Fig. S2).

**Fig. 5:**
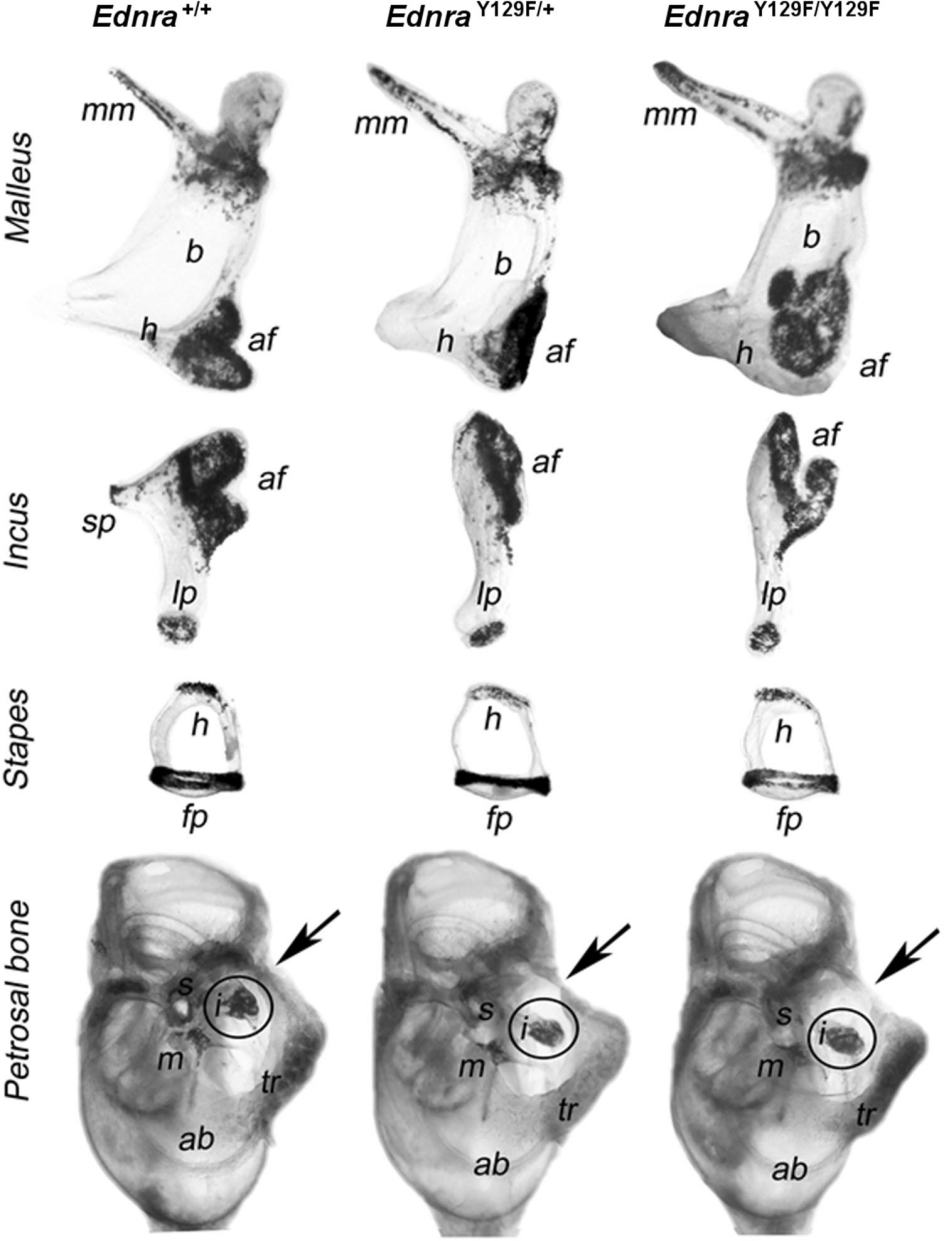
Middle and inner ears of *Ednra* ^+/+^ (*left*), *Ednra* ^Y129F/+^ (*middle*), and *Ednra* ^Y129F/Y129F^ (*right*) mice

Malformations of the malleus and incus were found by micro-dissections of the ossicles in adult mice. The malleal bodies appeared slimmer with planar-shaped facets in mutant mice, and stronger changes were seen in *Ednra* ^Y129F/Y129F^ mice. The short process of the incus was missing in both mutant mice. Dissection of the petrosal bone revealed abnormal closure of the ringbone and confirmed abnormally shaped incudo-malleal joints in the mutant mice (Fig. 5).

### Decreased lung volumetric parameters

Two of the MFDA patients showed upper airway obstruction necessitating tracheostomy (Gordon et al. 2015). In the micro-CT datasets of contrast-enhanced soft tissue of the upper airways, no clearly visible morphological changes were observed (data not shown). The lung function analysis showed that *Ednra* ^Y129F/+^ and *Ednra* ^Y129F/Y129F^ mice concomitantly exhibited decreased volumetric parameters in lung function analysis for IC, VC, ERV, and TLC with genotype-related severity for some parameters. In *Ednra* ^Y129F/Y129F^ mice, further to volumetric parameters FVC and forced expiratory volume (FEV 100) as flow parameters were significantly decreased. Additionally, one mechanical parameter, the Cchord, was decreased in *Ednra* ^Y129F/Y129F^ mice (Table 2).

**Table 2:** Significant volumetric parameter alterations following lung function analysis of female *Ednra* ^Y129F/+^ *, Ednra* ^Y129F/Y129F^, and *Ednra* ^+/+^ mice (mean ml ± SD)

	*Ednra* ^Y129F/+^	*Ednra* ^Y129F/Y129F^	*Ednra* ^+/+^
Volumetric parameters			
IC	0.967 ± 0.12 (*P* < 0.05)	0.775 ± 0.097 (*P* < 0.01)	1.138 ± 0.127
VC	1.32 ± 0.17 (*P* < 0.05)	1.08 ± 0.12 (*P* < 0.01)	1.56 ± 0.17
ERV	0.36 ± 0.06 (ns)	0.31 ± 0.04 (*P* < 0.01)	0.42 ± 0.05
TLC	1.295 ± 0.161 (ns)	1.112 ± 0.07 (*P* < 0.01)	1.496 ± 0.156
Flow parameters			
FVC	1.082 ± 0.141 (*P* < 0.05)	0.915 ± 0.109 (*P* < 0.01)	1.348 ± 0.149
FEV100	0.923 ± 0.122 (ns)	0.835 ± 0.086 (*P* < 0.01)	1.07 ± 0.097
Mechanical parameters			
Cchord^a^	0.085 ± 0.0105 (ns)	0.0683 ± 0.01 (*P* < 0.0098)	0.096 ± 0.012

*Cchord* static lung compliance, *ERV* expiratory reserve volume, *FEV*100 forced expiratory volume, *FVC* forced vital capacity, *IC* inspiratory capacity, *TLC* total lung capacity, *VC* vital capacity, *ns* not significant

^a^ (ml/cm H_2_O)

### Additional minor findings

Besides the observed major craniofacial abnormalities, we also found mild morphologic postcranial bone changes. Femoral metaphyseal and diaphyseal bone area in mice, BMC, and BMD measured at 3, 6, 9, and 12 months of age were slightly reduced in *Ednra* ^*Y129F/*+^ male and female mice but these reductions did not consistently reach statistical significance. Averaging values for males and females over all ages gave reductions of 6, 5, and 0 % for diaphyseal area, BMC, and BMD, respectively (Fig. S3). Similarly, metaphyseal area, BMC, and BMD were reduced 3, 5, and 1 %, respectively.

*Ednra* ^Y129F/Y129F^ mice appeared with a visible reduced body size, as observed in *Endra* ^−/−^ P0 mice (Clouthier et al. 1998), and also significantly reduced body weight (*P* < 0.001). Mean body weight was 23.58 ± 2.35 g in female and 28.31 ± 3.57 g in male *Ednra* ^Y129F/Y129F^ mice (female *Ednra* ^+/+^: 28.65 ± 3.21 g; male *Ednra* ^+/+^: 33.33 ± 3.41 g) in twelve-week-old mice. At the same age, in *Ednra* ^Y129F/Y129F^ but not in *Ednra* ^*Y129F/*+^ mice (*n* = 15 for all groups), body mass was significantly reduced in NMR measurement (*P* < 0.001).

We did not observe alterations in analyses of the allergy, eye, clinical–chemical, cardiovascular, immunology, neurology, nociception, pathology, and steroid metabolism screen. Parameters showing only mild tendencies to alterations were not considered. All phenotypic data are shown on the GMC webpage (www.mouseclinic.de) under the line code AEA001.

## Discussion

EDNRA has been shown to play a key role in craniofacial development (Clouthier et al. 2010; Twigg and Wilkie 2015). Detailed studies on the molecular regulation of craniofacial patterning were already performed using zebrafish, chick, and mouse embryos as targeted gene models (Miller et al. 2000; Ruest and Clouthier 2009; Zuniga et al. 2010). Here, we describe a new ENU mutagenesis-derived mouse model carrying a non-synonymous sequence variation within the *Ednra* gene leading to a replacement of the functionally important Tyr-129 residue within the transmembrane domain 2 of the receptor. In humans, the identical substitution is associated with the clinical spectrum of MFDA (Gordon et al. 2015), whereby alopecia is distinguishing this syndrome from other mandibulofacial dysostoses (MFD) (Wieczorek 2013). In *Ednra* ^Y129F^ mutant mice, numerous corresponding craniofacial characteristics such as dysplastic zygomatic arch with missing of the zygomatic process, micrognathia, dysplastic temporomandibular joints, typical changes of the orbital morphology, auricular dysmorphism, and conductive hearing loss were observed.

In addition to the abnormalities observed in patients with MFDA, we found volumetric airflow impairment in our mouse model. Breathing impairment in patients with MFDA was described due to upper airway obstruction without any closer specification (Gordon et al. 2015). We could not clearly detect any morphologic upper airway obstructions in our mouse model by soft-tissue contrast-enhanced micro-CT analysis so far. Nonetheless, female *Ednra* ^Y129F/+^ and *Ednra* ^Y129F/Y129F^ mice showed decreased volumetric parameters in lung function analysis. *EDNRA* caused human cellular airway smooth muscle proliferation when ligated to endothelin (Panettieri et al. 1996, 2008). In tracheostomized E18.5 *Ednra*-null pups, no changes of respiratory minute volume were observed but decreased ventilatory responses following breathing of hypoxic gas, suggesting that this observation was due to impaired early central respiratory control (Clouthier et al. 1998). Whether breathing impairment observed in MFDA-affected patients and in our mouse model may derive from upper or lower airway defects or both or are due to central defects is still unclear.

MFDA-affected individuals presented severe outer craniofacial malformations such as dysplastic zygomatic arch and mandible and jaw deformity confirmed by clinical CT scans of the skull. Broadening of the zygomatic bone together with missing of the squamosal zygomatic process was visible in some of the *Ednra* ^Y129F/+^ and *Ednra* ^Y129F/Y129F^ mice, in line with deformities of the temporomandibular joint. Three of the reported MFDA patients with heterozygous *de novo* mutations within the *EDNRA* gene were reported for conductive, but not specified, hearing loss (Gordon et al. 2015). For hearing impairment and cephalometric measurements in our mouse line, we found significant differences between *Ednra* ^+/+^, *Ednra* ^Y129F/+^, and *Ednra* ^Y129F/Y129F^ mice with a gene-dosage effect in mutant mice. More strikingly and in contrast to E18.5 *Ednra*-null embryos or newborn *Ece*-*1* ^−/−^ mice, in which the malleus and incus as well as the tympanic ring were completely missing (Clouthier et al. 1998; Yanagisawa et al. 1998), we found malleus and incus malformed, but almost normal stapes in *Ednra* ^Y129F/+^ and *Ednra* ^Y129F/Y129F^ mice. The endothelin signaling plays an important role in the development of skeletal elements that form the middle ear, involving the tympanic ring and the auditory ossicles (Ruest and Clouthier 2009). Malleus, incus, and tympanic ring are endochondral bones that derive from the first pharyngeal arch, while the stapes derive from the second pharyngeal arch (Mallo and Gridley 1996). Changes in the morphology and organization of the ossicles may impair the transmission of sound from the tympanic membrane to the cochlear oval window. We propose that the abnormal development of the tympanic ring affected the normal development of the middle ear cavity and tympanic membrane, thus causing aberrant three-dimensional positioning of the ossicles in the auditory bulla of our mice. We also hypothesize that the decreased acoustic startle reaction and PPI inhibition were secondary effects due to the hearing disability.

*Ednra* expression was shown for the first time in hair and vibrissal follicles of E15.5 *Ednra* ^lacz/+^ mouse embryos (Gordon et al. 2015). So far, no viable mouse model was available to study the impact of systemic impaired *Ednra* function on hair development in adult mice. Yet, we did not observe any alopecia in *Ednra* ^Y129F/+^ or *Ednra* ^Y129F/Y129F^ mice as reported for MFDA-affected patients. Inherited alopecia is correlated with a number of abnormalities including hormonal factors, alterations of gene transcription factors, impaired morphogenesis of hair follicles, and other cell signaling pathways (Tomann et al. 2016). However, how these and other underlying factors are exactly linked to healthy or aberrant morphology, hair follicle cycling, and subsequently hair loss is still unclear.

We further did not find any cleft palates in the analyzed mice of our cohort so far, as we could not observe any cleft palates in any of all our ENU mutagenesis-derived mouse models (930 mouse lines, unpublished data). Although cleft palates could be induced by triamcinolone in murine C3H strains when administered at E11.5 (Andrew et al. 1973), the C3H background probably is not sufficiently modeling human cleft palates (Juriloff and Harris 2008).

Edn-1 as a ligand of Ednra seems to be a key player for osteoblast differentiation by activating osteoblast signaling pathways, e.g., by activating Wnt signaling in bone (Clines et al. 2007), which additionally was shown by deletion of the Edn-1 receptor (Ednra) in osteoblasts leading to reduced bone mass (Clines et al. 2011). We observed only minor reductions in bone mass in male and female *Ednra* ^Y129F/+^ mice examined between 3 and 12 months of age. However, reductions in bone area and BMC were consistent with reduced body size and normal BMD indicating appropriately bone mass for slightly smaller bones, thus suggesting that the postcranial skeleton was not affected by the mutation.

However, we did not find any cleft palates, hypoplastic eyelids, alopecia, or dental abnormalities. As observed in MFDA-affected individuals, phenotypic outcome in our mouse model resulted in high individual variability of the phenotype. For some observations, a gene-dosage effect could be demonstrated by comparing *Ednra* ^Y129F/+^ with *Ednra* ^Y129F/Y129F^ mice.

So far, all available mouse models with a disruption of the *Ednra* gene were embryonically or perinatally lethal (Kurihara et al. 1994). In addition, no loss-of-function (LoF) mutation within the *EDNRA* gene was observed within the dataset of the human Exome Aggregation Consortium (ExAC Browser, www.exac.broadinstitut.org) providing exome sequencing data of 60,706 unrelated individuals of various disease-specific and population genetic studies. Instead of a complete loss of EDNRA such as in *Ednra*-null mice, an impaired *Ednra* function due to a change in ligand binding affinity caused by the Tyr-129 substitution (Krystek et al. 1994; Lee et al. 1994) leads to the phenotype in *Ednra* ^Y129F/+^ and *Ednra* ^Y129F/Y129F^ mice and patients with MFDA. Under physiological conditions, the two different ligands *Edn1* and *Edn2* bind to the G-protein-coupled *Ednra*, while all three ligands (Edn1–3) are binding to the endothelin B receptor (Ednrb) with specific affinities (Yanagisawa et al. 1998; Clouthier et al. 2010). We speculate that the substitution of Tyr-129 to Phe within the binding side of the ligand in EDNRA could have more than one effect, due to a reduction of Edn1/Edn2 receptor activation on one side and due to the gain of a physiological unknown activation of the receptor by Edn3 on the other side. The question of whether the Tyr129Phe exchange leads to a gain- or LoF mutation could not entirely be answered by the phenotypes observed in human patients and by zebrafish experiments (Gordon et al. 2015). Targeted inactivation of *Edn1*, *Ednra,* or *Ece1* in mouse embryos caused the transformation of lower jaw structures into maxillary derivatives (Abe et al. 2007), and vice versa, mice ectopically overexpressing *Edn1* showed an inverse transformation of the upper jaw into a mandible-like structure (Sato et al. 2008). Since the mutant EDNRA could rescue the phenotype in zebrafish lacking the endogenous *Ednra* genes, and further due to the malar abnormalities, possibly appearing as mandible-like structures, the non-synonymous sequence variation was suggested to be a gain-of-function mutation. Interestingly, in one of the *Ednra* ^*Y129F/*+^ mice analyzed in detail the structures of the zygomatic arch obviously still did exist. Thus, we suggest that the findings of micrognathia, morphologic changes of the ossicles, and gene-dosage-dependant hearing loss contribute more likely to a LoF mutation hypothesis. Nevertheless, the murine phenotype is, like the human and zebrafish phenotype, dependent on the complex context-dependent regulation of the EDNRA signaling via its physiological ligand during early craniofacial development. Inter-individual penetrance of the phenotype was observed in MFDA-affected individuals like in *Ednra* ^*Y129F/*+^ and *Ednra* ^Y129F/Y129F^ mice. Incomplete penetrance of the phenotype in autosomal dominant diseases may depend on genetic and environmental factors such as modifier genes, DNA sequence polymorphisms in regulatory elements, and random monoallelic expression of autosomal genes, where genes can be stably expressed, from either of the parental alleles (Cooper et al. 2013; Gendrel et al. 2016).

In conclusion, *Ednra* ^*Y129F*^ mice may serve as a valuable model to analyze endothelin signaling in syndromes resembling abnormalities of tissues derived from the first and second pharyngeal arches. We isolated the first viable mouse model for a systemic *Ednra* mutation and showed several bone abnormalities as observed in MFDA-affected individuals, but in more detail. Moreover, our mouse model could contribute to solve the so far open question on the functional nature of the mutation. Above all, our mouse model might be valuable for further analysis of symptoms observed in MFDA-affected individuals and for respective therapeutic interventions.

## Summary statement

ENU mutagenesis-derived *Ednra* ^Y129F^ mice mimic craniofacial phenotypes of jaw dysplasia, micrognathia, dysplastic temporomandibular joints, auricular dysmorphism, and missing of the squamosal zygomatic process as described for MFDA-affected individuals.

## Electronic supplementary material

Below is the link to the electronic supplementary material.

Supplementary material 1 (PDF 198 kb)

Supplementary material 2 (PDF 197 kb)

Supplementary material 3 (PDF 370 kb)

Supplementary material 4 (DOCX 24 kb)

Supplementary material 5 (MP4 2419 kb)

Supplementary material 6 (MP4 909 kb)

Supplementary material 7 (MP4 762 kb)
