# Supplementary material for: Viable EdnraY129F mice feature human mandibulofacial dysostosis with alopecia (MFDA) syndrome due to the homologue mutation
Source: Mamm Genome. 2016 Sep 26;27(11):587–98. doi: 10.1007/s00335-016-9664-5 (PMC5110705; doi:10.1007/s00335-016-9664-5)

Figure S1. Decreased acoustic startle reactivity in *Ednra*$^{Y129F/Y129F}$ mice.

Both male (A) and female (B) *Ednra*$^{Y129F/Y129F}$ mice show decreased acoustic startle reactivity compared to *Ednra*$^{+/+}$.

A

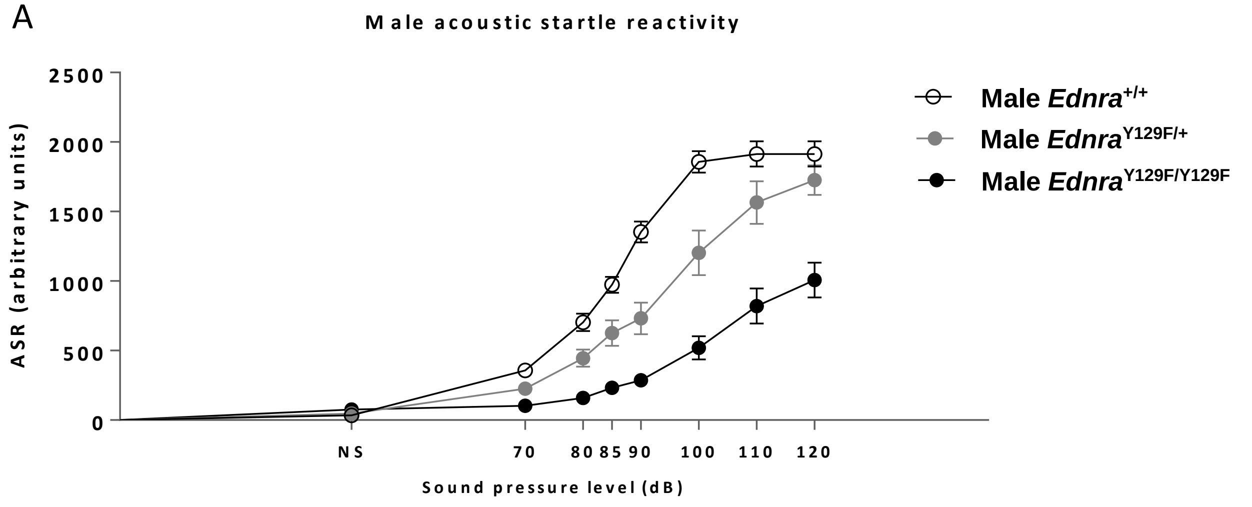

B

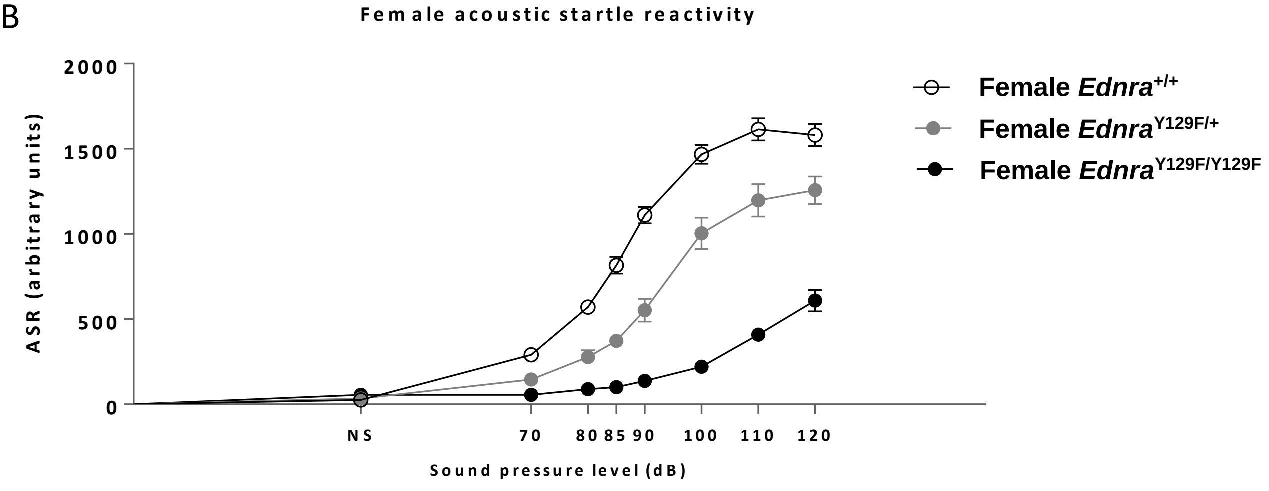

Supplement: Supplementary file 1 — Supplementary material 1 (PDF 198 kb) [file 335_2016_9664_MOESM1_ESM.pdf]
