# Supplementary material for: Viable EdnraY129F mice feature human mandibulofacial dysostosis with alopecia (MFDA) syndrome due to the homologue mutation
Source: Mamm Genome. 2016 Sep 26;27(11):587–98. doi: 10.1007/s00335-016-9664-5 (PMC5110705; doi:10.1007/s00335-016-9664-5)

Figure S2. Decreased prepulse inhibition in *Ednra*$^{Y129F/+}$ and *Ednra*$^{Y129F/Y129F}$ mice. Both male (A) and female (B) *Ednra*$^{Y129F/+}$ and *Ednra*$^{Y129F/Y129F}$ mice show decreased prepulse inhibition compared to *Ednra*$^{+/+}$ mice.

A

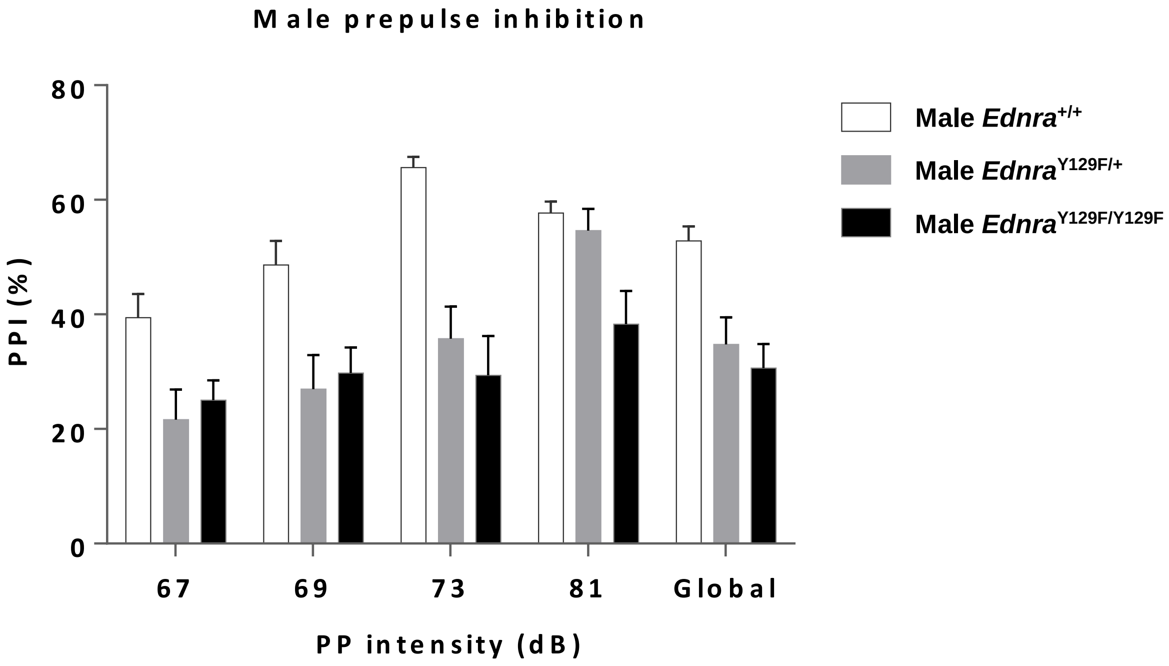

B

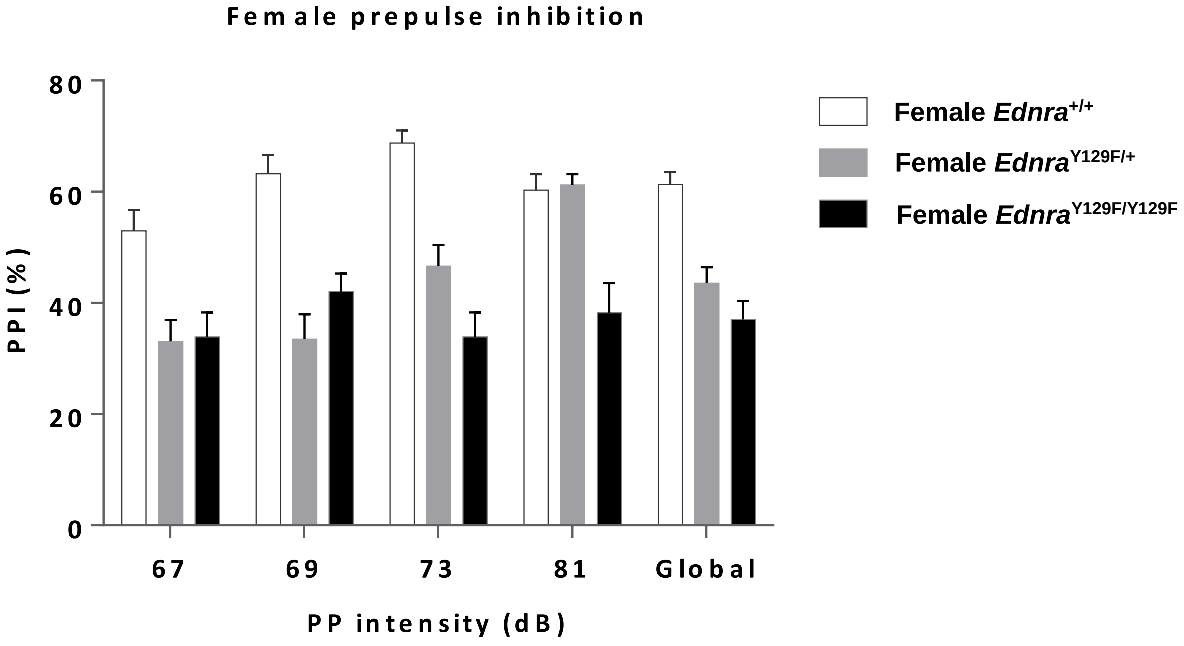

Supplement: Supplementary file 2 — Supplementary material 2 (PDF 197 kb) [file 335_2016_9664_MOESM2_ESM.pdf]
