# Supplementary material for: Viable EdnraY129F mice feature human mandibulofacial dysostosis with alopecia (MFDA) syndrome due to the homologue mutation
Source: Mamm Genome. 2016 Sep 26;27(11):587–98. doi: 10.1007/s00335-016-9664-5 (PMC5110705; doi:10.1007/s00335-016-9664-5)

Figure S3. pQCT measurement of bone parameters in three, six, nine and twelve months old *Ednra*$^{Y129F/+}$ mice.

Mean femur distal metaphyseal and midshaft diaphyseal bone area, BMC and BMD values for *Ednra*$^{Y129F/+}$ and *Ednra*$^{+/+}$ mice at three (n=10-12), six (n=7-13), nine (n=6-10) and twelve (n=5-11) months of age.

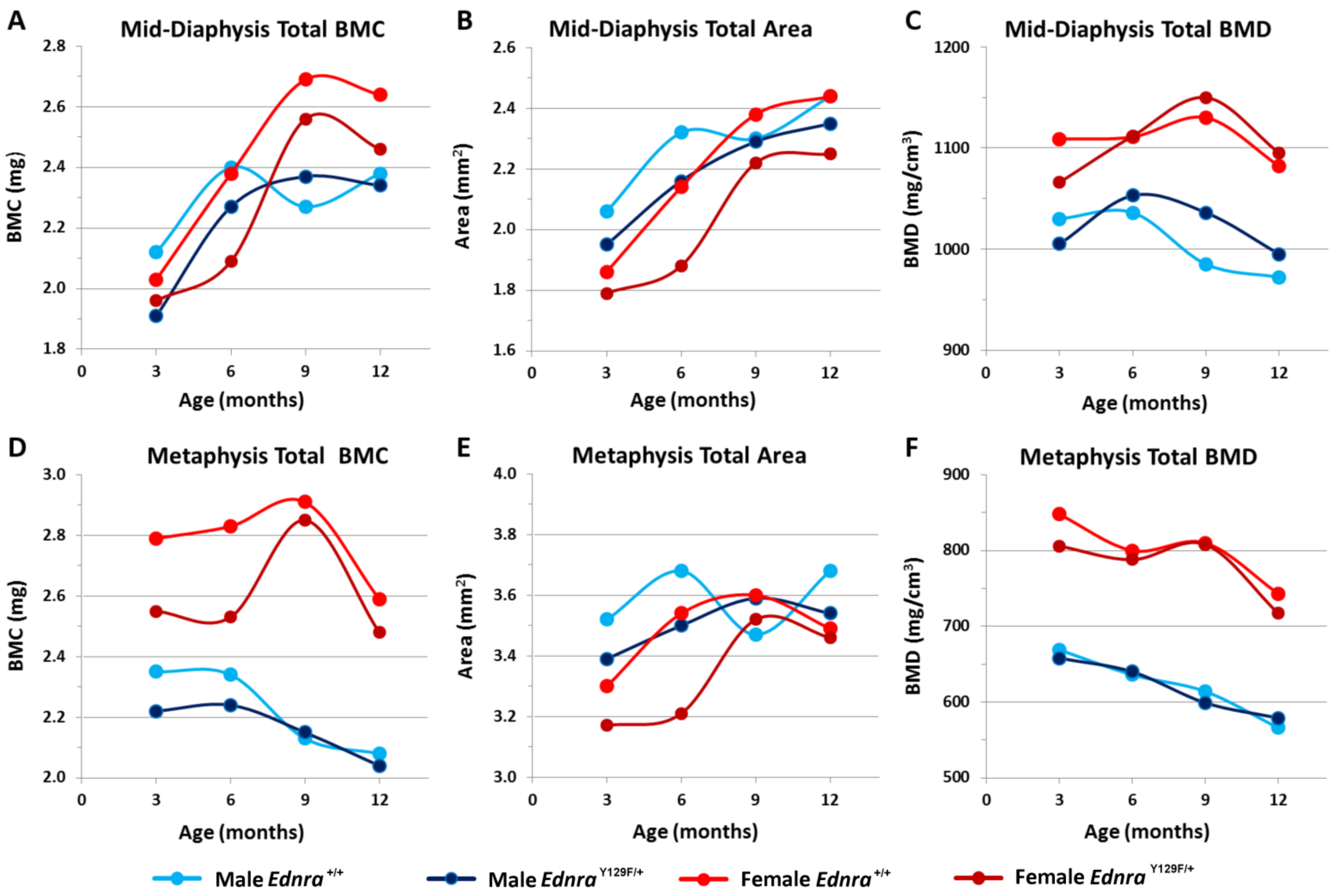

Supplement: Supplementary file 3 — Supplementary material 3 (PDF 370 kb) [file 335_2016_9664_MOESM3_ESM.pdf]
