# Supplementary material for: Viable EdnraY129F mice feature human mandibulofacial dysostosis with alopecia (MFDA) syndrome due to the homologue mutation
Source: Mamm Genome. 2016 Sep 26;27(11):587–98. doi: 10.1007/s00335-016-9664-5 (PMC5110705; doi:10.1007/s00335-016-9664-5)
Supplement: Supplementary file 4 — Supplementary material 4 (DOCX 24 kb) [file 335_2016_9664_MOESM4_ESM.docx]

**Supplementary Table S1. Methods and devices as used in the German Mouse Clinic for standardized phenotyping of *Ednra*^Y129F^ mice**

| **Screen** | **Methods** | **Age (weeks)** | ***Ednra*^+/+^**  **female/male** | ***Ednra*^Y129F/Y129F^ female/male** | ***Ednra*^Y129F/Y129F^**  **female/male** | | **References** |
| --- | --- | --- | --- | --- | --- | --- | --- |
|  |  |  |  |  |  |  | |
| **Behavior** | Open Field test | 8-9 | 9/10 | 10/10 | 10/10 | Mandillo et al. 2008 | |
|  | Prepulse Inhibition (PPI), Acoustic Startle Reflex (ASR) | 10-11 | 9/10 | 10/10 | 10/10 |  | |
|  |  |  |  |  |  |  | |
| **Cardiovascular** | Echocardiography (ECHO) | 14 | 9/10 | 10/13 | 10/7 | Sahn et al. 1978 | |
|  | Electrocardiogramm (ECG) | 14 | 9/10 | 10/13 | 10/7 | Desai et al. 1997; Mitchell et al. 1998 | |
|  |  |  |  |  |  |  | |
| **Clinical Chemistry** | Clinical chemical parameters measurement | 12 and 14  24  36  52 | 14/15  29/25  14/15  8/5 | 41/48  51/40  26/26  19/7 | 14/12  n.a.  n.a.  n.a. | Rathkolb et al. 2013; Sabrautzki et al. 2012 | |
|  | Hematological parameters | 12 and 14 | 14/15 | 15/18 | 14/12 | Fuchs et al. 2011 | |
|  | Non-esterified fatty acid concentration | 12 and 14 | 14/15 | 15/18 | 14/12 |  | |
|  | Glucose measurement ipGTT | 13 | 15/15 | n.a. | 15/18 | Neff et al. 2013 | |
|  | Carboxy-terminal collagen cross-link (CTX-1) measurement | 24  36  52 | 7/10  5/10  10/10 | 8/10  8/10  10/9 | n.a.  n.a.  n.a. | Sabrautzki et al. 2013 | |
| **Dysmorphology** | Peripheral quantitative computed tomography (pQCT) | 12  24  36  52 | 10/10  10/12  6/9  9/5 | 10/12  7/13  10/10  10/11 | n.a.  n.a.  n.a.  n.a. | Abe et al. 2006 | |
|  | Dual-energy X-ray absorptiometry (DXA) | 17 | 10/7 | n.a. | 10/10 | Abe et al. 2006 | |
| **Energy Metabolism** | Energy expenditure | 12 | 15/15 | n.a. | 15/15 | www.tse-systems.com | |
|  | Body composition | 12 | 15/15 | n.a. | 15/15 |  | |
| **Eye** | Scheimpflug imaging |  |  |  |  | Puk et al. 2013 | |
|  | Optical coherence tomography | 15 |  |  |  | Haller et al. 2010 | |
|  | Ocular parameters |  |  |  |  | Puk et al. 2006 | |
| **Neurology** | Auditory Brainstem Response (ABR) | 17 | 10/10 | 10/13 | 10/7 |  | |
|  | Gait analysis | 17 | 10/10 | 10/13 | 10/7 |  | |

n.a. = not analyzed

**Supplementary References**

Abe K, Fuchs H, Lisse T, Hans W, Hrabě de Angelis M (2006) New ENU-induced semidominant mutation, Ali18, causes inflammatory arthritis, dermatitis, and osteoporosis in the mouse. Mamm Genome 17, 915-926

Desai KH, Sato R, Schauble E, Barsh GS, Kobilka BK, Bernstein D (1997) Cardiovascular indexes in the mouse at rest and with exercises: new tools to study models of cardiac disease. Am J Physiol Heart Circ Physiol 272, H1053-1061

Haller F, Prehn C, Adamski J (2010) Quantification of steroids in human and mouse plasma using online solid phase extraction coupled to liquid chromatography tandem mass spectrometry. Nature Protocols 10.1038/nprot.2010.22

Mandillo S, Tucci V, Holter SM, Meziane H, Banchaabouchi MA et al. (2008) Reliability, robustness, and reproducibility in mouse behavioral phenotyping: a cross-laboratory study. Physiol Genomics 34, 243–255

Mitchell GF, Jeron A, Koren G (1998) Measurement of heart rate and Q-T interval in the conscious mouse Am J Physiol Heart Circ Physiol 274, H747-H751

Neff F, Flores-Dominguez D, Ryan DP, Horsch M, Schroeder S et al. (2013) Rapamycin extends murine lifespan but has limited effects on aging. J Clin Invest 123, 3272-3291

Puk O, Dalke C, Favor J, Hrabě de Angelis M, Graw J (2006) Variations of eye size parameters among different strains of mice. Mamm Genome 17, 851-857

Puk O, Hrabĕ de Angelis M, Graw J (2013) Lens tracking in mice by Scheimpflug imaging. Mamm Genome 24, 295-302

Rathkolb B, Hans W, Prehn C, Fuchs H, Gailus-Durner V et al. (2013) Clinical chemistry and other laboratory tests on mouse plasma or serum. Curr Protoc Mouse Biol 3, 101-119

Sahn DJ, DeMaria A, Kisslo J, Weyman A (1978) Recommendations regarding quantitation in M-mode echocardiography: results of a survey of echocardiographic measurements. Circulation 58, 1072-83
